# Bioactive Compounds for the Management of Hypertriglyceridemia: Evidence From Clinical Trials and Putative Action Targets

**DOI:** 10.3389/fnut.2020.586178

**Published:** 2020-11-30

**Authors:** Elisabetta Schiano, Giuseppe Annunziata, Roberto Ciampaglia, Fortuna Iannuzzo, Maria Maisto, Gian Carlo Tenore, Ettore Novellino

**Affiliations:** Department of Pharmacy, University of Naples Federico II, Naples, Italy

**Keywords:** hypertriglyceridemia, nutraceutical, food-derived bioactive compounds, cardiovascular risk, obesity

## Abstract

Hypertriglyceridemia refers to the presence of elevated concentrations of triglycerides (TG) in the bloodstream (TG >200 mg/dL). This lipid alteration is known to be associated with an increased risk of atherosclerosis, contributing overall to the onset of atherosclerotic cardiovascular disease (CVD). Guidelines for the management of hypertriglyceridemia are based on both lifestyle intervention and pharmacological treatment, but poor adherence, medication-related costs and side effects can limit the success of these interventions. For this reason, the search for natural alternative approaches to reduce plasma TG levels currently represents a hot research field. This review article summarizes the most relevant clinical trials reporting the TG-reducing effect of different food-derived bioactive compounds. Furthermore, based on the evidence obtained from *in vitro* studies, we provide a description and classification of putative targets of action through which several bioactive compounds can exert a TG-lowering effect. Future research may lead to investigations of the efficacy of novel nutraceutical formulations consisting in a combination of bioactive compounds which contribute to the management of plasma TG levels through different action targets.

## Introduction

Hypertriglyceridemia is defined as the presence of elevated concentrations of triglycerides (TG) in the bloodstream (TG >200 mg/dL) (1). Since these lipophilic molecules cannot travel freely within the blood, TG plasma concentration is represented by the sum of triglyceride-rich lipoproteins (TRLs), mainly, very-low-density lipoproteins (VLDL), chylomicrons and their remnants. Lipid disorders, including hypertriglyceridemia, are known to be associated with an increased risk of atherosclerosis, contributing overall to the onset of atherosclerotic cardiovascular disease (CVD) (2). Consequently, the management of lipid alterations for the prophylaxis of cardiovascular events is considered noteworthy. Guidelines for the reduction of plasma TG levels are based on both lifestyle intervention (e.g., decrease of body weight, reduction of dietary carbohydrates and alcohol intake, increased physical activity, etc.) and pharmacological treatment (1). However, it is well-known that poor adherence to lifestyle changes, medication-related costs, and side effects frequently associated with the pharmacological treatment, may limit the success of these interventions (3). Consequently, the search for alternative approaches to reduce plasma TG levels currently represents a hot research field. In this regard, an option for the management of hypertriglyceridemia may be represented by the supplementation with nutraceuticals, whose consumption is frequently associated with a good safety and tolerability profile (4).

The beneficial effects exerted on plasma lipid levels by numerous natural bioactive compounds are widely described in the scientific literature (5). Additionally, the metabolic targets through which these substances may improve TG levels seem to be different, suggesting a potential synergistic effect if administered in a properly combined way. The aim of this review is to summarize evidence about food-derived bioactive compounds involved in the management of plasma TG levels, with a specific focus on the data obtained from human studies and the putative action targets through which these natural substances may exert their beneficial effect.

## Search Strategy

For the section on the main putative targets of action for the TG-lowering effect, we focused on pre-clinical studies. A comprehensive literature search was conducted using the PubMed-NCBI database listings for relevant publications. The combinations of the following keywords were used: “nutraceuticals,” “natural compounds,” “bioactive compounds,” “cardiovascular risk,” “hypertriglyceridemia,” “dyslipidemia,” “mechanisms of action,” and “targets of action.” A thorough screening in scientific literature databases was conducted for the section on the evidence from clinical trials. We exclusively included (i) published studies from the last 5 years, (ii) studies investigating the effects of chronic administration with food-derived bioactive compound-based supplements, excluding both acute studies and diet-intervention studies, and (iii) studies reporting the effect of exclusive nutraceutical supplementation, excluding those investigating the administration with pharmaceutical treatments.

## Evidence From Clinical Trials

Although not directly investigating TG-reducing effects, several studies have reported the ability of such food-derived bioactive compounds to modulate lipid metabolism-related biomarkers, including serum TG. In various studies, these observations derived from investigation on diverse subjects, with or without pathological conditions, and, in many cases, within the evaluation of different outcomes. However, a modest number of studies described marked and significant TG-reducing effects, providing evidence for individuation of such effective phytochemicals for designing nutraceutical formulations. In this section we summarize the most relevant clinical trials reporting the TG-reducing effect of food-derived bioactive compounds (listed and detailed in Table 1), including omega-3 (6–33), niacin (34, 35), *Trigonella foenum-graecum L*. (36, 37), fiber (38–45), vitamin E (46, 47), and polyphenols (48–61).

**Table 1:** Most relevant published clinical trials from the last 5 years reporting TG-reducing effect after chronic supplementation with food-derived bioactive compounds.

**Trial type**	**Subjects**	**N^**°**^ of subjects**	**Age (years)**	**Lifestyle intervention**	**Treatment(s)**	**Duration of treatment**	**Serum TG variation (%)**	**References**
Omega-3
R, DB, PC	Obese adolescents with hypertriglyceridemia	65	12.5 ± 1.8	Controlled diet and physical activity	• Soybean oil 3,000 mg/d (placebo) • PUFAs 3,000 mg/d (containing 2,000 mg EPA and 1,000 mg DHA)	12 weeks	−44.1 (*p* < 0.01) compared to baseline	Huang et al. (6)
R, DB, PC, PA	Obese adolescents with hypertriglyceridemia	130	10–16	*n.a*.	• Soybean oil 3,000 mg/d (placebo) • PUFAs 3,000 mg/d (containing 2,000 mg EPA and 1,000 mg DHA)	12 weeks	−39.1 (*p* < 0.01) compared to baseline	Del-Río-Navarro et al. (7)
R, DB, PC	Obese adults with T2DM	81	57 ± 2.2	Controlled diet and physical activity	• Placebo • Fish-oil • Curcumin • Curcumin + fish oil	12 weeks	−16.5 ± 4.5 (*p* = 0.007) compared to baseline	Thota et al. (8)
R, OL	Overweight adults with hyperlipidemia	96	48.7 (active group) 50.7 (dietary fish group)	*n.a*	• OM-3 2 g (containing 180 mg EPA and 120 mg DHA in each soft-gel) • 250 g farmed trout fish	2 months	−30.7 (*p* = 0.021)	Zibaeenezhad et al. (9)
R, DB, PC,	Obese adults with NAFLD	176	55.3 ± 13.3 (active group) 55.1 ± 10.9 (placebo group)	*n.a*	• Olive oil (placebo) • OM-3 concentrate MF4637	24 weeks	−18.0 (*p* = 0.0008) compared to baseline	Tobin et al. (10)
R, DB, PC	Adults with schizophrenia and MetS	65	12.5 ± 1.8 (active group) 12.7 ± 10.5 (placebo group)	*n.a*	• Vitamin E 100 mg/d • OM-3 group received fish oil containing 720 mg EPA and 480 mg DHA	12 weeks	−27.1 (*p* < 0.01) compared to baseline	Xu et al. (11)
R	Children with ERDS	49	13.33 ± 2.44	*n.a*	• OM-3 two capsules/d containing 360 mg EPA and 240 mg DHA	3 months	−30.4 (*p* < 0.001) compared to baseline	Omar et al. (12)
R, DB, PA, PC	Adults with T2DM	30	50.47 ± 6.06 (active group) 50.67 ± 6.70 (placebo group)	*n.a*.	• OM-3 capsules (containing 180 mg EPA and 120 mg DHA) • Placebo (gelatin capsules)	8 weeks	−17.0 (*p* = 0.005) compared to baseline	Fayhn et al. (13)
R, SC, DB	Adults with chronic Chagas cardiomyopathy	42	58.6 ± 11.0 (active group) 55.0 ± 9.5 (placebo group)	Controlled diet	• OM-3 capsules (containing 1.8 g EPA and 1.2 g DHA) • Placebo (corn oil)	8 weeks	−25.9 (*p* < 0.01) compared to baseline	Silva et al. (14)
Mc, Pr, OL, SA	Adults with T2DM and high risk for cardiovascular diseases	12	65 ± 11	*n.a*.	• OM-3 4 g/d (containing 1,860 mg EPA and 1,500 mg DHA)	12 weeks	−12.1 (*p* < 0.001)	Ide et al. (15)
R, DB, PC	DN	60	62.9 ± 10.5 (active group) 62.4 ± 9.6 (placebo group)	*n.a*.	• OM-3 1,000 mg/d • Placebo	12 weeks	−19.8 ± 8.8 (*p* = 0.01) compared to baseline	Soleimani et al. (16)
R, SB, PC	Adults with IR	45	56.8 ± 3.09 (active group) 58.2 ± 15.19 (placebo group)	*n.a*.	• Paraffin oil (placebo) • OM-3 1,800 mg/d (6 soft-gel capsules, each containing 180 mg EPA and 120 mg DHA)	4 months	−17.2 (*p* = 0.02) compared to baseline	Gharekhani et al. (17)
R, DB, PC, PA	Adults with modest hypercholesterolaemia and hypertriglyceridaemia	260	51.6 ± 11.5 (active group) 51.0 ± 10.0 (placebo group)	*n.a*. (usual diet)	• Fish oil EPA + DHA + 2 PS 1 g/d • Placebo	4 weeks	−10.6 (*p* < 0.001) compared to baseline	Blom et al. (18)
R, DB, PC,	Women with obesity	57	45.9 ± 9.3 (active group) 47.3 ± 12.0 (placebo group)	*n.a*. (usual diet and physical activity)	• 3 capsules, each containing 90–150 mg EPA and 430 mg DHA • Placebo	3 months	−17.6 (*p* = 0.002) compared to baseline	Polus et al. (19)
R, DB, PC	Adults with severe hypertriglyceridemia	273	51 ± 10 (active group OM3-CA 2 g/d) 53 ± 11 (active group OM3-CA 4 g/d) 51 ± 11 (placebo group)	*n.a*.	• OM3-CA 2 g/d • OM3-CA 4 g/d • Olive oil (placebo)	12 weeks	OM3-CA 2 −15.79 (*p* < 0.001) compared to baseline OM3-CA 4 −22.76 (*p* < 0.001) compared to baseline	Morton et al. (20)
R, DB, PC,	Overweight adults with hypertension, dyslipidemia, diabetes, or smoking	40	30–74	*n.a*.	• OM-3 2 g/d • Placebo	2 months	−25.3 (*p* < 0.001) compared to baseline	Barbosa et al. (21)
IS, PC	Adults with dyslipidemia	14	40.2 ± 1.7	*n.a*.	• OM-3 fatty acid ethyl esters 4 g/d (containing 1,860 mg/d EPA ethyl ester and 1,500 mg/d DHA)	4 weeks	−47.9 (*p* = 0.003) compared to baseline	Furuhashi et al. (22)
R, CO, DB, Co	Obese adults with MetS	154	53.5 ± 14	*n.a*.	• EPA (2.7 g/d), • DHA (2.7 g/d) • Corn oil (placebo)	10 weeks	EPA = −13.3 (*p* < 0.0001) DHA = −11.9 (*p* < 0.0001) compared to baseline	Allaire et al. (23)
R, DB, PC	Adults with T2DM	85	50.93 ± 7.27	*n.a*	• 3 softgels of OM-3 (each containing 600 mg EPA and 300 mg DHA) • 3 softgels of placebo (each containing 900 mg of edible paraffin)	10 weeks	−18.0 (*p* < 0.056) compared to baseline	Mazaherioun et al. (24)
R, DB, Mc, PC	Postmenopausal women with a history of stage I to III hormone–sensitive breast cancer	249	59.5 (active group) 59.1 (placebo group)	*n.a*.	• 6 capsules/d (each containing 560 mg EPA + DHA in a 40/20 ratio) • Blends soybean and corn oil (placebo)	12 weeks	−22.1 (*p* < 0.001) compared to baseline	Hershman, et al. (25)
R	Adults	191	26.6 ± 6.3	*n.a*.	Fish oil 2.7 g/d	6 weeks	−5.3 (*p* < 0.05) compared to baseline	Binia et al. (26)
R, GC	Obese adults	26	48.6 ± 6.8 (active group) 49.3 ± 4.8 (placebo group)	*n.a*.	ALA	12 weeks	−51.8 (*p* < 0.0001) compared to baseline	Zhao et al. (27)
R, DB, PC,	Adults with ESRD and high cardiovascular risk	161	66 ± 11 (active group) 68 ± 11 (placebo group)	*n.a*.	• OM-3 1.7 g/d • Olive oil (placebo)	3 months	−10.7 (*p* < 0.01) compared to baseline	Sørensen. et al. (28)
R, DB, PC	Adults with NASH	78	59.4 ± 7.2 (active group) 52.6 ± 6.6 (placebo group)	*n.a*. (Usual diet)	• 50 mL PUFAs with 1:1 ratio of EHA and DHA • Prescribed normal saline (placebo)	6 months	−28.0 (*p* = 0.01) compared to baseline	Li et al. (29)
R, DB, PC	Adults with IR and T2DM	68	55.8 ± 7.6 (active group) 56 ± 7 (placebo group)	*n.a*. (Usual diet)	• Softgels containing 600 mg OM-3 (362.5 mg DHA and 100 mg EPA) • Paraffin softgels (placebo)	2 months	−25.6 (*p* = 0.01) compared to baseline	Toupchian et al. (30)
R, DB, PC	Children with NAFLD	51	11.0 ± 2.6 (active group) 10.8 ± 2.8 (placebo group)	*n.a*	• 39% DHA algae oil • 290 mg linoleic acid supplied with germ oil (placebo)	6 months	−18.5 (*p* = 0.04) compared to baseline	Pacifico et al. (31)
R, DB, PC,	Adults with T2DM	63	30–70	*n.a*.	• DHA-rich fish oil (containing 2,400 mg/d fish oil; DHA: 1,450 mg and EPA: 400 mg) • Placebo	8 weeks	−49.3 (*p* = 0.003) compared to baseline	Mansoori et al. (32)
R, DB, PC, CO	Adults with T2DM	10	54.7 ± 7.6	*n.a*.	• Fish oil 5 g/d, containing 3 g of EPA (64%) and DHA (36%) • 5 g/d of corn and soybean oil (placebo)	6 weeks	−9.7 (*p* = 0.05) compared to baseline	Tremblay et al. (33)
Niacin
R, Mc, DB, PC	Subjects with or without CKD	3,413 (CKD = 505; No CKD = 2,908)	70.7 ± 7.3 (CKD group) 62.5 ± 8.4 (No CKD group)	*n.a*.	• Niacin 1,500 mg • Placebo	3 years	−36.0 (CKD group) and−26.5 (no CKD group) after 1 year (*p* < 0.001 compared to placebo); −35.42 (CKD group) and −27.10 (No CKD group) after 3 years (*p* < 0.001 compared to placebo)	Kalil et al. (34)
R, DB, PC, CO	Hypertriglyceridemic patients	8	18–65	*n.a*.	• Placebo • Increasing dose of niacin (0.5 to 2.0 g/d) in the first four weeks and 2.0 g/d for the last four weeks	8 weeks	−46 (*p* = 0.023) compared to placebo	Croyal et al. (35)
***Trigonella foenum-graecum L***.								
R, Co	Newly diagnosed T2DM patients	95	Range not clearly defined	*n.a*.	• Control group not clearly defined • *Trigonella foenum-graecum* seed powder 50 mg/d	1 month	−23.53 (*p* < 0.001) compared to baseline	Geberemeskel et al. (36)
OL, SA	Hyperlipidemic and hyperglycaemic patients	25	56 ± 8	*n.a*.	• Polyherbal formulation 12.8 g/d containing 39.1% (5 g) of *Trigonella foenum-graecum* seed powder	40 days	−18.22 (*p* < 0.01) compared to baseline	Zarvandi et al. (37)
**Fiber**								
R, DB, PA, PC	Overweight and obese adults	93	19–68	*n.a*. (usual diet)	• Psyllium 5 g/d (PSY) • PolyGlycopleX (PGX) 5 g/d • Rice flour 5 g/d (placebo)	12 months	−12.7 (*p* = 0.023) in PSY group at 6 months compared to baseline	Pal et al. (38)
SC, R, DB, PC	Overweight and obese children	38	7–12	*n.a*.	• Oligofructose-enriched inulin 8 g/d • Maltodextrins 3.3 g/d (placebo)	16 weeks	−19.08 (*p* = 0.024) compared to baseline	Nicolucci et al. (39)
R, DB, PC	Overweight and obese adults with MetS	87	20–65	*n.a*.	• Inulin 6 g/d in a fortified yogurt • Fiber-free yogurt (placebo)	10 weeks	−32.65 (*p* < 0.001, compared to baseline; *p* = 0.003, compared to placebo)	Mohammadi-Sartang et al. (40)
R, PC	Type 2 diabetics	22	50–80	*n.a*.	• Functional bread (7 g fiber/100 g and 7.62 g β-glucans/100 g) • Normal bread (3.2 g fiber/100 g and 0 g β-glucans/100 g)	6 months	−15.7 (*n.s*.) compared to baseline	Tessari et al. (41)
R, DB, PC	Overweight and obese adults	26	31.3 ± 8.5	Energy-restricted diet (−500 kcal/d)	• Yacon flour 25 g/d	6 weeks	−10.63 (*n.s*.) compared to baseline	Machado et al. (42)
R, DB, PC	Type 2 diabetics	91	50.09 ± 9.3	*n.a*. (usual diet and physical activity)	• Gum Arabic 30 g/d • Pectin 5 g/d (placebo)	3 months	−11.03 (*p* = 0.061) compared to baseline	Babiker et al. (43)
R, PC, TB	Women with PCOS	62	18–45	*n.a*.	• Resistant dextrins 20 g/d • Maltodextrins 20 g/d (placebo)	3 months	−2.63 (*p* = 0.008) compared to baseline; −38.50 (*p* = 0.001) compared to placebo	Gholizadeh Shamasbi et al. (44)
R, PC, TB	Women with T2DM	60	30–65	*n.a*. (usual diet and physical activity)	• Resistant starch 10 g/d • Maltodextrins 10 g/d (placebo)	8 weeks	−9.90 (*p* < 0.05) compared to baseline	Gargari et al. (45)
**Vitamin E**								
R, DB, PC	Women with PCOS	86	20–40	*n.a*.	• Vitamin E 400 IU/d • CoQ10 200 mg/d • Vitamin E 400 IU/d + CoQ10 200 mg/d • Placebo	8 weeks	Vitamin E group: −5.82 (n.s. compared to baseline; *p* < 0.001 compared to placebo)	Izadi et al. (46)
R, CO, PC	Haemodialysis patients	37	50.7 ± 16.5 (active group) 47.9 ± 21.5 (placebo group)	*n.a*. (usual diet)	• 30 mL virgin argan oil (containing 44 mg vitamin E/100g) • Placebo	4 weeks	−13.23 (*p* < 0.01 compared to placebo)	Eljaoudi et al. (47)
**Polyphenols**								
R, SA, DB	Adults with MetS	78	62 ± 9	*n.a*.	• 2 pills/d, containing 160 mg of Curcuma longa, 102 g of silymarin, 24 mg of guggul lipids, 14 mg of chlorogenic acid, and 2.5 mg of inulin	4 months	−8 (*p* = 0.059)	Patti et al. (48)
R, DB, PC	Obese subjects with high serum TG (> 200 mg/dL)	45	40–80	Healthy diet, rich in fruits and vegetables and poor in fats and carbohydrates (1,200 kcal/d)	• Bergamot polyphenols 650 mg/d • Polyphenols 1,300 mg/d • Maltodextrins (placebo)	3 months	−32 (*p* < 0.0001 compared to placebo)	Capomolla et al. (49)
R, DB PC, CO	Pre-hypertensive male	60	24–72	*n.a*.	• 20 mL olive leaf polyphenolic extract (including 6.81 mg oleuropein/mL; 0.32 mg hydroxytyrosol/mL; 0.12 mg tyrosol/mL) • Placebo	6 weeks	−12.16 (*p* < 0.05) compared to baseline	Lockyer et al. (50)
R, PC	Patients with NAFLD	77	46.3 ± 11.5 (active group) 48.9 ± 9.7 (placebo group)	*n.a*.	• Curcumin 500 mg/d • Placebo	8 weeks	−13.12 (*p* = 0.05) compared to baseline	Rahmani et al. (51)
R, DB, PC	Patients with nephropathy	40	38.5 ± 11.1 (active group) 35.4 ± 12.0 (placebo group)	*n.a*.	• Resveratrol 500 mg/d + 500 mg curcumin/d • Placebo	12 weeks	−38.25 (*p* = 0.01) compared to baseline and placebo	Murillo Ortiz et al. (52)
R, DB, PC, Mc	Dyslipidemic patients	98	30–65	Healthy diet with exercise at least 4 days/week	• Amla extract (containing 350 mg polyphenols) 1000 mg/d • Roasted rice powder 1000 mg/d (placebo)	12 weeks	−34.15 (*p* < 0.0001) compared to baseline	Upadya et al. (53)
R, DB, PC	Type 2 diabetics	44	40–70	*n.a*. (usual diet and physical activity)	• Curcumin 1,500 mg • Rice flour (placebo)	10 weeks	−12.09 (*p* < 0.05) compared to baseline	Adibian et al. (54)
R, PA, DB, PC	Type 2 diabetics	20	45–65	*n.a*. (usual diet and physical activity)	• Green tea polyphenolic extract 400 mg/d • Calcined magnesia (placebo)	12 weeks	−38.48 (*n.s*.) compared to baseline	Quezada-Fernández et al. (55)
R, DB, PA, PC	Adults	56	25–65	Controlled diet	• Cranberry juice 480 mL • Placebo	8 weeks	−10.15 (*p* < 0.05) compared to placebo	Novotny et al. (56)
R, DB, PC	Type 2 diabetics	43	30–60	*n.a*.	• Resveratrol 480 mg/d • Starch 480 mg/d (placebo)	4 weeks	−8.16 (*n.s*.) compared to baseline	Zare Javid et al. (57)
R, DB, PC	Schizophrenia patients	19	18–65	Controlled diet	• Resveratrol 200 mg/d • Placebo	4 weeks	−11.44 (*n.s*.) compared to baseline	Zortea et al. (58)
R, DB, PC	Patients with NALFD	50	44.0 ± 10.1 (active group) 46.3 ± 9.5 (placebo group)	Controlled energy-balanced diet and physical activity	• Resveratrol 500 mg/d • Placebo	12 weeks	−20.55 (*n.s*.) compared to baseline	Faghihzadeh et al. (59)
Pr, SA, DB	Adults with moderate hypercholesterolemia	80	55 ± 13	*n.a*.	• Bergamot derived extract at a fixed dose daily (150 mg of flavonoids, with 16% of neoeriocitrin, 47% of neohesperidin and 37% of naringin)	6 months	−17 (*p* = 0.002) compared to baseline	Toth et al. (60)
R, DB, Pc	Adults with T2DM	58	58.1 ± 2.3 (active group) 57.6 ± 3.4 (placebo group)	*n.a*.	• Anthocyanins 320 mg/d • Placebo	24 weeks	−23 (*p* < 0.01) compared to placebo	Li et al. (61)

*ALA, α-linolenic acid; CA, carbossilic acids; Co, controlled; CO, crossover; CVD, cardiovascular disease; DB, double-blind; DHA, docosahexaenoic acid; DM, diabetes mellitus; DN, diabetic nephropathy; EPA, eicosapentaenoic acid; ESRD, end-stage renal disease; hsPCR, High-Sensitivity C-Reactive Protein; IPE, icosapent ethyl; IR, insulin resistance; Mc, multicenter; MetS, metabolic syndrome; n.a., not-applicable; n.s., non-significant; NAFLD, non-alcoholic fatty liver disease; NASH, non-alcoholic steatohepatitis; OL, open-label; OM-3, omega-3; PA, parallel arm; PC, placebo-controlled; PCOS, polycystic ovary syndrome; PS, plant sterol; Pr, prospective; PUFAs, polyunsaturated fatty acids; R, randomized; SA, single-arm; SB, single-blind; SC, single center; T2DM, type 2 diabetes mellitus; TB, triple-blind; TG, triglyceride*.

## Main Putative Targets of Action for the Reduction of Plasma Triglyceride Levels

### Influence on Fatty Acid Beta-Oxidation

Fatty acid beta-oxidation is a metabolic process taking place in the mitochondrial matrix by which fatty acids (FAs) are broken down into acetyl-CoA units, with the release of energy in the form of adenosine triphosphate (ATP). The main regulation of this aerobic process occurs at the level of carnitine palmitoyltransferase I (CPT1), which is in turn inhibited by high concentrations of malonyl-CoA, the first intermediate in FA synthesis. For this reason, FA catabolism and FA synthesis represent opposite metabolic pathways that cannot occur simultaneously (62). Plasma TG levels in humans are represented by the sum of the TG content in TG-rich lipoproteins (TRLs), mainly, postprandial chylomicrons and VLDL. Being plasma free fatty acids (FFA) one of the main sources for TRL synthesis (63), reduced availability of these substrates through the improvement of FA oxidation may represent a useful strategy, in order to achieve a decrease in plasma TG levels. Moreover, in a study conducted by Vega et al. (64), moderately obese patients with hypertriglyceridemia showed lower levels of 3-hydroxybutyrate (regarded as fatty acid oxidation marker) compared with normotriglyceridemic control group, suggesting that an impaired FA oxidation process may lead to an increase of plasma TG levels. Based on these observations, the study of bioactive compounds which act by improving FA degradation, appears currently noteworthy. In this regard, peroxisome proliferator-activated receptor alpha (PPARα) represents a key regulator of lipid metabolism, enhancing FA oxidation process and reducing fat storage (65). PPARα is one of the three subtypes of ligand-inducible transcription factors belonging to the superfamily of PPARs. The transcription factor PPARα is considered a sensor of endogenous lipids and it is found in organs subjected to lipid catabolism, such as liver, skeletal muscle, kidneys, heart, and brown fat (66). The improved expression of this nuclear receptor has several effects in the liver, including an up-regulation of the transcription of genes involved in peroxisomal and mitochondrial oxidation of FAs, such as acyl-CoA oxidase, CPT-I, and CPT-II (67).

The main pharmacological treatment that shows PPARα as a target of action is based on fibrates (68), drugs currently recommended for the management of hypertriglyceridemia by the European Society of Cardiology (ESC) and the European Atherosclerosis Society (EAS) (1). In the group of bioactive substances capable to affect TG metabolism through a PPARα-mediated pathway, a growing interest is focused on the supplementation with omega-3 fatty acids. Omega-3 and omega-6 fatty acids represent two categories of polyunsaturated fatty acids (PUFAs), whose consumption through diet is considered essential, due to the mammalian lack of ability to introduce double bonds into FAs. Fish is the main source of long-chain omega-3 FAs, mainly eicosapentaenoic acid (EPA) and docosahexaenoic acid (DHA), while some plant-based foods (such as flaxseed oil, soy and canola oil, chia seeds, pumpkin seeds and walnuts) contain omega-3 in the form of precursor alpha-linolenic acid (ALA) (69). PPARα is well-recognized to be directly bound and activated by omega-3, leading to the induction of genes involved in FA oxidation (70). An *in vitro* study also showed that the 9-oxo-10(E),12(Z),15(Z)-octadecatrienoic acid (9-oxo-OTA), an active compound deriving from ALA occurring in tomato fruit extract, was able to activate PPARα and induce mRNA expression of PPARα target genes in murine hepatocytes (71). Overall, based on this mechanism of action, and through other mechanisms described below, omega-3 may allow to address liver lipid metabolism toward a state of oxidation, rather than synthesis or accumulation.

It is still a matter of debate whether ALA itself mediates the effects on lipid metabolism and inflammation or if these beneficial effects are mediated by EPA and DHA. In this regard, a recent 12-week RCT investigated the effects of diets enriched with lean fish and fatty fish (animal source of omega-3) or *Camelina sativa* oil (vegetable source of omega 3 in the form of precursor ALA) in subjects with impaired fasting glucose. At the end of the study, the beneficial effects of fatty fish and *Camelina Sativa* oil on lipid metabolism were confirmed, while the increased concentrations of anti-inflammatory lipid mediator appeared to be associated with the intake of both plant and animal-based omega-3 PUFAs (72). Another evidence derives from a study conducted on healthy subjects with moderate dyslipidemia, in which the effect of a nutraceutical formulation, based on gastro-resistant capsules containing cryo-micronized chia seeds and vitamin E, was tested (73). Among plant-based sources of omega-3, chia (*Salvia hispanica L*.) is of major interest, not only as regards its high content of precursor ALA, but also for its omega-3:omega-6 FA ratio (~4:1) and its high content of dietary fiber, minerals, vitamins (e.g., niacin), and polyphenolic compounds (74). Considering the significant beneficial effects on plasma TG levels observed after 8 weeks of supplementation (−27.5%; *p* = 0.0095), this study provides further demonstration of ALA-mediated beneficial effects on lipid metabolism. Although omega-6 and omega-3 series are both essential, the balance of these FAs in the diet is critical. Based on the analysis of available literature, it was suggested an omega-6:omega-3 ratio of ~6:1, which results instead unbalanced in favor of omega-6 PUFAs in the typical Western diet, reaching a ratio of 15:1 or even greater. Since the long chain derivatives of omega-6 and omega-3 PUFAs depend on the same enzymes for their production (75), the efficiency of endogenous conversion of ALA into EPA and DHA is limited by the presence of high levels of omega-6 in the Western diet. In parallel, higher intake of omega-6 leads to an increased production of linoleic acid (LA) metabolites (such as arachidonic acid, ARA) compared to ALA metabolites (such as EPA and DHA). This has important practical implications in relation to the profoundly different biological effects of LA and ALA metabolites, especially for their inflammatory and anti-inflammatory activity, respectively (76).

Recently, curcumin, the active principle of turmeric (*Curcuma longa*), has gained a greater attention by scientific research as a potential agent for the management of hyperlipidemia (77). As regards the effects exerted on TG metabolism, curcumin was suggested to positively regulate FA beta-oxidation through the up-regulation of PPARα expression (78). PPARα was also reported to be directly bound and activated by astaxanthin, a carotenoid particularly abundant in seafood (e.g., kill oil), which demonstrated to reduce TG concentration in HepG2 cells through a PPARα-mediated mechanism of action (79). Furthermore, results from *in vitro* studies on bioactive compounds of tea suggested the regulation of PPARs subtypes as a putative mechanism of action that may lead to the hypotriglyceridemic effects observed *in vivo*. As previously reviewed (80), the activation of PPARα by epigallocatechin-3-gallate (EGCG) of tea would take place through an indirect mechanism of action. Blocking of the inhibitor of kB phosphorylation induced by EGCG may in fact determine the reduced activation of the nuclear transcription factor-kB (NF-kB). Since NF-kB may counteract the PPARα-mediated expression of genes involved in FA oxidation, its inhibition allows to avoid this undesirable interaction (81). In contrast, a dose-dependent direct interaction with the PPARα ligand-binding domain was reported in the case of linalool, an aromatic terpenoid found in tea (82).

Finally, among the natural bioactive compounds which could decrease plasma TG levels *via* PPARα, polyphenols play an important role, with particular regard to the class of flavonoids. For instance, cyanidin has proven to be a moderate PPARα ligand (83), while recent evidence has highlighted that proanthocyanidins from a grape seed extract could indirectly act on PPARα expression through the *in vitro* and *in vivo* inhibition of the activity of histone deacetylase class I (HDACs), mainly, of HDAC-3. Since the latter is a well-recognized repressor of PPARα expression (84), its inhibition can lead to an increase in PPARα phosphorylation and activation, resulting in an improvement of its targeted gene expression (85).

### Reduction of Free Fatty Acid Flux to the Liver

Obesity is known to potentially contribute to the onset of several metabolic alterations, including hypertriglyceridemia, resulting in an overall increased risk of CVD (86). The increase in plasma TG levels may be related to an alteration of adipose tissue function. This alteration, called as “adiposopathy,” often occurs in the presence of visceral fat accumulation and can lead to a rise of intra-adipocyte lipolysis process (87). Free fatty acids (FFA) derived from adipose tissue lipolysis are subsequently released into the circulation and transported to the liver by hepatic artery and portal vein, causing an increase in the synthetic rate of TG (63). Hormone-sensitive lipase (HSL) is well-recognized as the key enzyme for TG mobilization, mainly, in adipose tissue and skeletal muscle, with an opposite function to that the lipoprotein lipase (LPL) (88). Among the TG-lowering agents, nicotinic acid (also known as niacin or vitamin B3), has proven to exert lipid-regulating effects when administered at high doses (1.5–6 g/d) (89). Although the mechanism of action of nicotinic acid in the management of lipid profile remains unclear, the potential of this organic compound to reduce the expression of HSL has been described (90). In addition, the niacin-mediated inhibition of HSL expression would occur more specifically in peripheral fat (91), where HSL seems to exert a major lipolytic capacity (92). Overall, the inhibition of this lipase may result in a decrease of FAs output from the adipose tissue, a reduced delivery of these molecules to the liver, and the consequent inhibition of hepatic TG synthesis.

### Inhibition of Fatty Acid Synthesis

As described above, the main regulator of FA beta-oxidation is CPT1, which contributes to the transport of long-chain FA into the mitochondrial matrix. CPT1 is allosterically inhibited by high concentrations of malonyl-CoA, the first intermediate in the biosynthesis of FA. For this reason, FA catabolism and FA synthesis are opposite metabolic pathways which cannot occur simultaneously (62). The first enzyme involved in FA synthesis, and consequently in the intermediate production of malonyl-CoA, is acetyl CoA carboxylase (ACC). In turn, the activity of this enzyme results to be suppressed by the phosphorylation induced from adenosine monophosphate-activated protein kinase (AMPK), an essential regulator of energy metabolism (93). In this scenario, bioactive compounds with AMPK-activating capacity, represent potential candidate to help direct lipid metabolism toward a state of oxidation, rather than synthesis or accumulation. In this regard, as recently reviewed by Vazirian et al. (94), different bioactive compounds (e.g., curcumin, quercetin, resveratrol, berberine, ginsenosides and EGCG), demonstrated to activate AMPK, thus, contributing to the inhibition of ACC and FA synthesis.

Sterol-regulatory-element-binding protein 1c (SREBP-1c), together with the PPARs nuclear receptors, is recognized as one of the main transcription factors for the regulation of lipid metabolism. The activation of several FA synthase genes is regulated by this transcription factor, such as ACC, fatty acid synthase (FAS), and stearoyl-CoA desaturase (SCD). Therefore, the inhibition of SREBP-1c may lead to the lack of conversion of acetyl-CoA into FAs and the consequent process of lipogenesis (95). The expression of the nuclear receptor SREBP-1c is widely reported to be inhibited by PUFAs, but not from monounsaturated or saturated FAs (95, 96). Among PUFAs which can affect the activity of SREBP-1c, omega-3 play a major role. The hypotriglyceridemic effect of omega-3 is well-recognized, deriving not only from an increased lipolysis, as described above, but also from a reduction in the lipogenesis process. In particular, genes involved in the lipogenesis process (e.g., FAS, SCD1, and Δ6-desaturase) contain a sequence responsive to omega-3 in the promoter region, to which the transcription factor SREBP-1c binds (97). Moreover, different studies suggest that omega-3 may reduce SREBP-1c activity also through the proteasome-mediated degradation of this transcription factor (98, 99). Altogether, these effects lead to a reduced synthesis of fatty acids and triglycerides, followed by the lack of formation of nascent VLDL particles. Interestingly, it has been shown that the expression, as well as the nuclear transcription of SREBP-1c, could be also inhibited by dietary curcumin (100, 101).

The inhibition of SREBP-1c activity, as a putative mechanism of action which may contribute to the reduction of lipid synthesis, is also described in a study performed by Vijayakumar et al. (102). The authors conducted an *in vitro* study on HepG2 cells in which a down-regulation of SREBP-1c expression, both at the mRNA and protein level, was observed after treatment with a thermostable extract of fenugreek seeds. This effect was accompanied by a decreased expression of FAS enzyme and, consequently, a decrease in lipid synthesis. It is important to underline that, at a molecular level, the expression of SREBP-1c is suppressed by the farnesoid X receptor (FXR), another important transcription factor, belonging to nuclear receptors superfamily. This interaction is mediated by a signaling pathway involving small heterodimer partner (SHP) (103). This observation strongly suggests the use of substances capable of improving the activity of FXR, in order to obtain a plasma TG-lowering effect. Interestingly, results deriving from an *in vitro* study, showed the potential of procyanidins occurring in a grape seed extract in modulating positively FXR activity, enhancing the natural binding of this transcription factor by bile acids (104), and determining the *in vivo* reduction of plasma TG content. In addition, an *in vitro* study conducted by Zhao et al. (105) reported that PUFAs are selective modulators of FXR, specifically regarding ALA, DHA, and ARA. Since FXR plays a critical role in lipid metabolism, the interaction of PUFAs with this transcription factor was suggested as a further mechanism of action which may contribute to their beneficial effects on lipid profile.

Finally, an additional mechanism of action potentially involved in the inhibition of hepatic lipogenesis is the reduction of activity of TG-synthesize key enzymes, mainly, diacylglycerol O-acyltransferase (DGAT) and phosphatidic acid phosphatase (PAP). PAP is an enzyme which catalyzes the conversion of phosphatidic acid into diacylglycerol, while DGAT1 and DGAT2 are two enzymes which catalyze the last step in the biosynthesis of TG, both in the glycerol phosphate and monoacylglycerol pathways (106). Several studies suggest the beneficial effects of different bioactive compounds on lipid metabolism via the inhibition of these two enzymes, including omega-3 and nicotinic acid (107–111).

### Increased Clearance of Plasma Triglyceride

Plasma TG levels are represented by the TG content of TRLs, mainly, postprandial chylomicrons and VLDL. Chylomicrons are the largest lipoprotein particles (about 1,000 nm) and are composed of TG (85–90%), phospholipids, cholesterol esters and 1–2% of different types of apolipoprotein (apoB48, apoCIII, apoCII, and apoE). These exogenous lipoproteins are formed in the small intestine after the ingestion of dietary fats and subsequently enter the blood circulation through the lymphatic system. In the vascular endothelium, the apoCII present on the surface of the chylomicrons activates the LPL which favors the lipolysis of TG. FFA and chylomicron remnants will result from this hydrolyzing process: the first products (FFA), based on individual metabolic needs, can be used by muscle cells as a source of energy or resynthesized into TG and stored in adipose cells, while the second products (chylomicron remnant particles) are removed from the circulation by the liver. Dietary fats are not the only source of TG, but can also be synthesized in liver cells and, together with apoB100, apoCIII, apoCII, and apoE, form VLDL particles, which are released into the bloodstream (112).

One of the putative strategies to obtain a plasma TG-lowering effect is represented by the supplementation with substances capable to increase the clearance of plasmatic TRLs. In this regard, LPL is considered a key enzyme for TG clearance and is known to be regulated by several factors. Particularly, apoCII represents an essential co-factor for LPL activation, whereas apoCIII may inhibit its action and, consequently, the resulting lipolysis process (113). In addition, apoCIII may contribute to the onset of hypertriglyceridemia through the stimulation of VLDL synthesis and the inhibition of hepatic remnants uptake (114). In this scenario, omega-3, whose use as a food supplement is widely extended for the management of TG levels, showed to act also through the increased LPL activity. Dietary long-chain omega-3 PUFAs have demonstrated to direct up-regulate LPL gene expression and PPARγ mRNA expression in cultured adipocytes (115, 116), and the agonism of this latter transcription factor may further contribute to enhance the LPL activity (117). Moreover, results obtained from an *in vivo* study showed a significant reduction in plasma TG levels in mice fed a high-fat diet, with the source of fat represented by omega-3 from fish oil, compared to other three diets (soy oil, a mix of soy oil and fish oil and a normal chow diet). Based on the obtained data, it was suggested that plasma TG-lowering effect, observed in the group of mice fed with fish oil, could depend on the increased LPL activity and, consequently, on the improved clearance of TRLs induced by omega-3 (118). Besides, the reduction of apoCIII plasma levels, as a mechanism that lead to an increased TRL cycle, was also observed in humans after the supplementation with EPA and DHA combined, or purified EPA (119, 120). Evidence from scientific literature also suggest a putative action of FXR in down-regulating apoCIII gene expression and up-regulating apoCII and VLDL-receptor gene expression (121–123). This observation may additionally support the hypothesis that bioactive compounds, capable of interacting with FXR, could exert beneficial effects on TG metabolism. Furthermore, as reviewed by Giglio et al. (124), polyphenols have demonstrated to reduce plasma TG levels via increasing the activity of LPL. In this context, flavonoids, phenolic acids (cinnamic acid), curcumin and sylimarin are the polyphenols with the most demonstrated beneficial efficacy (124).

Lipid metabolism is known to be also influenced by oxidative stress: an increase of oxidative state of the organism may contribute to lipid peroxidation, and this process may influence the activity of hepatic lipases, especially regarding LPL (125). As a proof of this, a positive correlation between the LPL activity and the serum total antioxidant capacity (TAC) of the organism was described (126). Based on this observation, two *in vivo* studies on streptozocin-induced diabetic rats showed the efficacy of a high supplementation with vitamin E in bringing TG back to normal levels. Considering the well-known antioxidant potential of vitamin E, these studies propose the efficacy of vitamin E in removing lipid peroxides in plasma and liver, as the potential mechanism that may lead to an increased activity of LPL (127, 128). In addition, a study performed on triton-treated hyperlipidemic rats showed a decrease in plasma TG levels after chronic feeding with fenugreek seed extract at a dosage of 200 mg/kg body weight. This lipid-lowering effect was accompanied by the activation of different hepatic lipases, including LPL, and by the concomitant reduction of plasma lipid peroxidation, as observed *in vitro* (129). However, these preliminary results should be deepened thoroughly, especially by performing clinical trials. Nevertheless, it is also important to underline the widely recognized importance of antioxidant dietary compounds, especially vitamin E, in counteracting the onset of diseases related to both liver fat accumulation and oxidative stress, such as non-alcoholic fatty liver disease (NAFLD) (130, 131).

### Inhibition of Triglyceride Intestinal Absorption

The absorption of TG across the intestinal mucosa requires the conversion of these molecules from insoluble macroscopic particles into soluble micelles, which can be specific targets of intestinal enzymes. In this regard, the main enzyme responsible for the hydrolysis of total dietary fats is pancreatic lipase, which converts TG into diglycerides, monoglycerides, FFA, and glycerol (132). Orlistat, a drug approved by the Food and Drug Administration (FDA) for obesity treatment, acts as an inhibitor of pancreatic lipase enzyme activity and its administration also promotes the reduction of plasma TG levels in humans (133). In this scenario, interest in the use of natural bioactive compounds, capable of inhibiting pancreatic lipase, is increasingly emerging. The inhibition of pancreatic lipase by plant-derived molecules was recently reviewed by Rajan et al. (134), with a specific focus on the role of flavonoids, alkaloids, saponins and terpenoids. Among these bioactive compounds, the class of flavonoids showed the most powerful inhibiting activity on pancreatic lipase enzyme, and this potential effect was mainly dependent on molecule weight and position of hydroxyl groups (129). However, investigation of new classes of bioactive compounds with this potential beneficial activity is extensive and still ongoing (135).

Dietary fibers are other significant food components capable to counteract the absorption of postprandial TG. It is known that fiber can reduce the absorption of fats in the small intestine, thus decreasing the production of TRLs, mainly chylomicrons (136). In particular, *in vitro* studies have shown that dietary fibers may affect lipid metabolism mainly through two different mechanisms of action: (1) soluble fibers are capable of creating viscous solutions in the gastrointestinal tract delaying lipid absorption and transit in the small intestine (137); (2) other fibers, such as chitosan, can directly bind lipid globules, with the final result of inhibiting lipolysis (138). At this regard, animal-based studies with chitosan supplementation have demonstrated the ability of this biopolymer in reducing plasma TG levels, supporting a chitosan derived reduction in lipid absorption (139). Nonetheless, different studies showed that dietary fiber, such as pectin, can inhibit lipase enzyme activity (140–142). Overall, research on bioactive substances potentially capable of reducing the absorption of dietary fats, may be considered as an interesting strategy for the management of plasma TG levels, especially during the postprandial state.

## Conclusion

**Figure 1:**
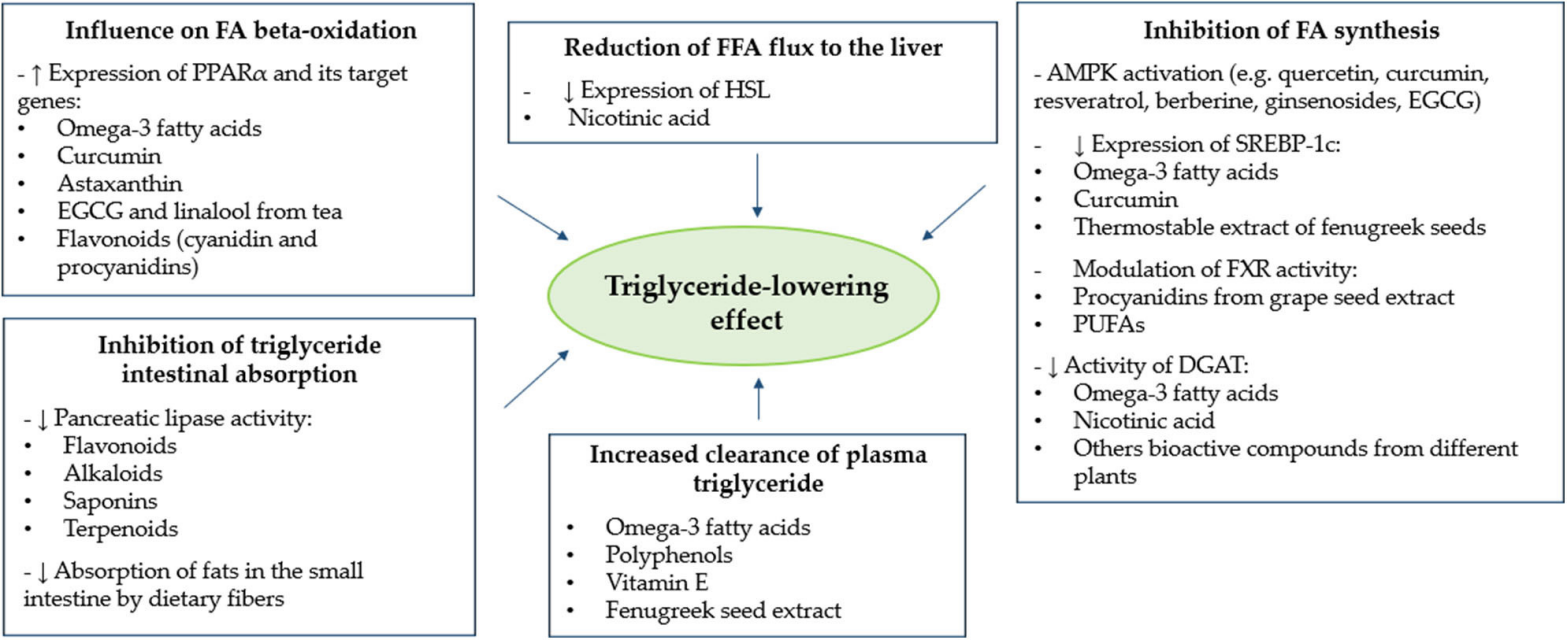
Main putative action targets of different bioactive compounds for the reduction of plasma triglyceride levels. AMPK, adenosine monophosphate-activated protein kinase; DGAT, diacylglycerol O-acyltransferase; EGCG, epigallocatechin-3-gallate; FA, fatty acid; FFA, free fatty acid; FXR, farnesoid X receptor; HSL, hormone-sensitive lipase; PPARα, peroxisome proliferator-activated receptor alpha; PUFAs, polyunsaturated fatty acids; SREBP-1c, sterol-regulatory-element-binding protein 1c.

In the present review, we summarized the most relevant evidence about the TG-reducing effect of different food-derived bioactive compounds. In this regard, after analysis of the available scientific evidence, we have identified omega-3, niacin, vitamin E, *Trigonella foenum-graecum L*, fibers and polyphenols, as agents with a demonstrated plasma TG-lowering effect after a chronic supplementation in humans. Moreover, analysis of data obtained from *in vitro* studies allowed us to describe and classify the main putative targets of action through which several bioactive compounds may exert a beneficial effect on plasma TG metabolism. As shown in Figure 1, such action targets include (a) influence on FA beta-oxidation, (b) reduction of FFA flux to the liver, (c) inhibition of FA synthesis, (d) increased clearance of plasma TG, and (e) inhibition of TG intestinal absorption. The increase of FA beta-oxidation and the inhibition of FA synthesis represent two different and opposite targets of action which, in both cases, may lead to an increased rate of FA oxidation, rather than synthesis or accumulation. Since TG molecules are composed of FAs, it is reasonable to hypothesize that the effects of food-derived bioactive compounds on these two targets may lead to a final TG-lowering effect. Furthermore, two other putative metabolic targets are the reduction of FFA flux to the liver and the increased clearance of plasma TG. In both cases, the effect of bioactive compounds described above would be mediated by the reduced and increased activity of HSL and LPL, respectively. Plasma TG can origin from FAs provided from both endogenous and exogenous sources (112). The inhibition of intestinal absorption of TG represents the last target of action described in this review, and it refers, specifically, to the improved metabolism of TG from exogenous sources (dietary food).

To our knowledge, this is the first report focusing on the description and categorization of putative mechanisms of action through which different natural bioactive substances may determine, in a directly or indirectly manner, a TG-reduction effect. Notably, the TG metabolic targets of nutraceuticals may be different, and the same substance can exert a beneficial effect at distinct levels. It could be hypothesized that the appropriate combination of different bioactive compounds may determine an additional, or even synergistic, beneficial effect on plasma TG levels in humans. The investigation of these possible synergistic actions currently represents an interesting line of research. More scientific evidence, deriving from well-designed intervention studies, are needed to deepen this aspect.

## Author Contributions

All authors listed have made a substantial, direct and intellectual contribution to the work, and approved it for publication.

## Conflict of Interest

The authors declare that the research was conducted in the absence of any commercial or financial relationships that could be construed as a potential conflict of interest.
